# Risk factors for mental health disorders during the COVID-19 pandemic in Spain: A cohort study

**DOI:** 10.1093/eurpub/ckac129.265

**Published:** 2022-10-25

**Authors:** A Miranda-Mendizabal, J Piqueras, P Castellví, S Álvarez, S Díaz, L Gómez, S Recoder, N Sánchez, E García Durán, CG Forero

**Affiliations:** 1 School of Medicine, Universitat Internacional de Catalunya, Sant Cugat del Vallès, Spain; 2 Department of Basic Sciences, Universitat Internacional de Catalunya, Sant Cugat del Vallès, Spain

## Abstract

**Background:**

Data comparing the populations’ mental health from before, during and after the pandemic is needed. We aim to assess the risk factors for the first-onset and persistence of major depressive disorder (MDD) and suicidal thoughts and behaviours (STB) during the first year of the pandemic among the Spanish general population.

**Methods:**

Cohort study through two online surveys from before the pandemic (N = 2,005, October/November 2019) and 12-months later (N = 1,357) on an adult Spanish, nationally representative, population-based sample. Multiple logistic regression models were used to assess the association between socio-demographic, COVID-19 related variables and healthcare received during the pandemic with the onset and persistence of MDD and STB.

**Results:**

Women have more than 3-fold risk for the onset (OR 3.18; CI95% 1.40 -7.22) and persistence (OR 8.62; CI95% 1.74-42.48) of MDD. Studying and working at the same time (OR 10.13; CI95% 2.17-47.35) and having close relatives/friends with COVID-19 infection (OR 14.84; CI95% 1.91-115.18) or death (OR 5.26; CI95% 1.56-17.73) due to COVID-19 are risk factors for MDD onset. Sick-leave (OR 17.19; CI95% 2.65-112.56) and unemployment (OR 7.01; CI95% 1.85-26.43) increased the risk for MDD persistence. Death of friends/colleagues due to COVID-19 (OR 8.40; CI95% 1.47-48.07) increased the risk for STB onset, and being on sick-leave (OR 7.91; CI95% 1.80-34.66) for STB persistence.

**Conclusions:**

During the COVID-19 pandemic, women were consistently more at risk of having worse mental health than men. Direct and indirect consequences caused or aggravated by the pandemic are common risk factors for the increased risk for the onset and persistence of both MDD and STB. Identification of high-risk subgroups and risk factors for MDD and STB among the Spanish general population will allow the developing and implementing of evidence-driven strategies for reducing the long-term impact of the pandemic in populations’ mental health.

**Key messages:**

• The pandemic consequences, whether due to having had COVID-19, having close people affected or who have died from the infection and the social consequences increase the risk for worse mental health.

• Evidence-driven strategies for reducing the long-term impact of the pandemic in populations’ mental health should be a public health priority.

